# Acute radiotherapy-associated oral pain may promote tumor growth at distant sites

**DOI:** 10.3389/fonc.2023.1029108

**Published:** 2023-05-19

**Authors:** Constanza S. Meneses, Emily M. Gidcumb, Karen L. Marcus, Yarines Gonzalez, Yen Hao Lai, Santosh K. Mishra, B. Duncan X. Lascelles, Michael W. Nolan

**Affiliations:** ^1^ Department of Clinical Sciences College of Veterinary Medicine, North Carolina State University, Raleigh, NC, United States; ^2^ Comparative Medicine Institute, North Carolina State University, Raleigh, NC, United States; ^3^ Translational Research in Pain, College of Veterinary Medicine, North Carolina State University, Raleigh, NC, United States; ^4^ College of Veterinary Medicine, North Carolina State University, Raleigh, NC, United States; ^5^ Department of Molecular Biomedical Sciences, College of Veterinary Medicine, North Carolina State University, Raleigh, NC, United States; ^6^ Comparative Pain Research and Education Center, North Carolina State University, Raleigh, NC, United States; ^7^ Thurston Arthritis Center, The University of North Carolina School of Medicine, Chapel Hill, NC, United States; ^8^ Center for Translational Pain Research, Department of Anesthesiology, Duke University, Durham, NC, United States; ^9^ Duke Cancer Institute, Duke University, Durham, NC, United States

**Keywords:** pain, radiotherapy, sensory nerves, cancer, mouse, TRPV1

## Abstract

**Introduction:**

Patients developing acute radiotherapy induced dermatitis or oral mucositis commonly experience pain. When severe, this radiotherapy-associated pain (RAP) can necessitate treatment breaks; unfortunately, in a variety of cancers, prolongation of the radiotherapy course has been associated with early cancer relapse and/or death. This is often attributed to accelerated repopulation, but it is unknown whether pain or pain signaling constituents might alter tumor behavior and hasten metastatic disease progression. We studied this by testing the hypothesis that severe acute RAP at one site can hasten tumor growth at a distant site.

**Methods:**

Mice underwent single fraction tongue irradiation (27 Gy, or 0 Gy “sham” control) to induce severe glossitis. At the time of maximal oral RAP, one of three luciferase-transfected tumor cell lines were injected *via* tail vein (4T1, B16F10, MOC2; each paired to their syngeneic host: BALB/c or C57BL/6); tumor burden was assessed *via in vivo* transthoracic bioluminescence imaging and *ex vivo* pulmonary nodule quantification. Survival was compared using Kaplan-Meier statistics.

**Results:**

Tongue irradiation and resultant RAP promoted lung tumor growth of 4T1-Luc2 cells in BALB/c mice. This effect was not a result of off-target radiation, nor an artefact of environmental stress caused by standard (subthermoneutral) housing temperatures. RAP did not affect the growth of B16F10-Luc2 cells, however, C57BL/6 mice undergoing tail vein injection of MOC2-Luc2 cells at the time of maximal RAP experienced early lung tumor-attributable death. Lung tumor growth was normalized when RAP was reduced by treatment with resiniferatoxin (300 µg/kg, subcutaneously, once).

**Discussion:**

This research points towards radiation-induced activation of capsaicin-responsive (TRPV1) neurons as the cause for accelerated growth of tumors at distant (unirradiated) sites.

## 1 Introduction

In patients undergoing radiotherapy, treatment course prolongation can negatively impact prognosis (1). Unplanned extension of the time over which radiotherapy is administered has been associated with diminished locoregional tumor control and reduced progression-free survival rates (2–4). In one study of patients with head and neck squamous cell carcinoma (HNSCC), a 10-day prolongation of the overall treatment time was associated with a 10% reduction in tumor control probability (5). Others have estimated a 4.8% higher risk of local relapse per day of interruption (6). From a mechanistic perspective, increased risk for early relapse (despite definitive irradiation) has been attributed to accelerated repopulation, which is a phenomenon whereby sublethal doses of radiation stimulate tumor clonogen proliferation *via* activation of the receptor tyrosine kinase, epidermal growth factor receptor (7).

Clinically, a common reason for treatment course prolongation is acute radiation-associated pain (RAP). Acute and potentially severe RAP frequently accompanies radiodermatitis and oral mucositis (8, 9). This pain can be difficult to manage (10), and so to improve patient comfort, unplanned treatment breaks may be recommended by the physician to allow tissue healing before completing the prescribed course of radiotherapy. Thus, pain can prompt treatment course prolongation, which adversely affects patient prognosis, but it is unknown whether this pain (or pain signaling mechanisms) might alter tumor behavior to directly increase the risk for early relapse at either the primary tumor site, or at distant metastatic sites.

In other realms of cancer research, it has been demonstrated that certain mediators of pain can promote aggressive tumor cell behaviors. For example, human breast tumor biopsies display widespread immunoreactivity for nerve growth factor (NGF, a prototypical neurotrophic factor and well-recognized molecular mediator of pain), and in xenografted mice, NGF blockade inhibits both tumor growth and metastasis (11). For similar reasons, anti-NGF therapy has been suggested as a potential strategy for improving comfort and slowing tumor growth in human oral cancer patients (12). In addition, strategies to reduce pain can also alter cancer progression. It has been shown by Page and colleagues that after tail vein injection of tumor cells into rats, growth of neoplastic lung nodules is hastened by the presence of surgical pain; this effect is mitigated with opioid analgesics (13, 14). The premise that, for certain cancers, tumor control may be maximized *via* optimization of intra- and perioperative analgesia and anesthesia is further supported by clinical observations. Use of spinal anesthesia as an alternative to general anesthesia has been associated with lower recurrence rates after transurethral resection of superficial bladder cancers (15). Overall survival was longer in a series of patients having undergone open thoracotomy for primary lung tumors with paravertebral blocks instead of intravenous patient-controlled analgesia (16).

These reports provide a rational basis for considering the possibility that pain, or pain signaling mechanisms, may contribute to poor oncologic outcomes in patients undergoing therapeutic irradiation. Here, we adapted the methods of Page and colleagues, to explore (in animal models) if aggressive tumor behavior may be a direct result of severe RAP, or RAP signaling components. Specifically, we set out to test the hypothesis that lung tumor growth would be accelerated in mice undergoing tail vein injection of tumor cells while they had severe acute RAP, and that this effect could be reversed with effective analgesic therapy.

To generate and model the effects of acute RAP, our experiments relied upon high dose single-fraction tongue irradiation protocol that reliably induces severely painful glossitis (17). We began by establishing that in female mice, growth of lung tumors after tail vein injection of an aggressive mammary carcinoma cell line is hastened by the presence of severe acute orofacial RAP. Next, we established that this effect is attributable to local oral irradiation (*vs*. non-target radiation or certain environmental stressors), and is seen in both male and female mice, but is restricted to certain tumor models. Finally, we demonstrate that the effect can be mitigated with provision of effective analgesia by selectively chemically ablating a subset of sensory neurons that express the transient receptor potential vanilloid type-I receptor (TRPV1).

## 2 Materials and methods

### 2.1 Animals

Ten- to fourteen-week-old BALB/c (female and male) and C57BL/6 (female) mice were purchased from a commercial vendor (Charles River Laboratories). Mice were group-housed (2-4 animals/cage), and had unrestricted access to water, food pellets, and nutritional gel (DietGel 76A, Clear H2O) in a controlled 12-hours day-night cycle. Studies were performed at room temperature (21-22°C) unless otherwise noted. Pre-defined criteria for euthanasia included: increased respiratory effort, anorexia for more than 24 hours (once glossitis had resolved), hunched posture, or more than 30% weight loss. Euthanasia was performed *via* asphyxiation (3.5 L/min CO_2_ chamber), followed by cervical dislocation. All experimental procedures were approved by the Institutional Animal Care and Use Committee at North Carolina State University (protocol #’s: 18-061-B and 19-810-B).

### 2.2 Cell lines

Three different firefly luciferase-expressing mouse cancer cell lines were used: 4T1-Luc2 (mammary carcinoma), B16F10-Luc2 (cutaneous melanoma), and MOC2-Luc2 (oral squamous cell carcinoma). The 4T1-Luc2 cells were cultured in Dulbecco’s modified eagle medium (DMEM, high in glucose with sodium pyruvate and glutamine, Fisher Scientific) with 10% fetal bovine serum (Genesee Scientific) and 500 µg/mL of zeocin (InvivoGen). The B16F10-Luc2 cells cultured in RPMI 1640 (Gibco) containing 10% fetal bovine serum (Genesee Scientific). The media was supplemented with 100 units/mL penicillin and 100 μg/mL streptomycin (Genesee Scientific). The MOC2-Luc2 cells were cultured in HyClone™ Iscove’s Modified Dulbecco’s Medium (IMDM) and Hams F12 Nutrient Mixture (Fisher Scientific) at a 2:1 mixture with 5% fetal bovine serum, 1% penicillin/streptomycin, 5 ng/mL epidermal growth factor, 400 ng/mL hydrocortisone, and 5 mg/mL insulin (MilliporeSigma). All cells were incubated at 37°C in 5% CO_2_ atmosphere and passaged 1:10 when 75-90% confluent. Cell lines underwent authentication *via* short tandem repeat analysis and PCR-based interspecies contamination testing performed by a third-party purveyor (CellCheck Mouse 19; IDEXX), and also underwent routine Mycoplasma testing.

### 2.3 Irradiations

Within each experiment, mice were randomly allocated to one of two treatment groups: 1) single-dose irradiation (27 Gy) of the rostral tongue to induce severe but reversible glossitis (hereafter referred to as ‘‘IR” mice) or 2) sham irradiation (0 Gy) as the negative control (referred to as ‘‘SHAM” mice). In some experiments we also used total body irradiation (1.6 Gy; referred to as ‘‘TBI” mice) to simulate the non-target dose the body is exposed to when using the aforementioned 27 Gy protocol. Irradiations were performed using a clinical linear accelerator and 6 MV X-ray beam (Novalis TX, Varian Medical Systems). A detailed description of these irradiation protocols, including all associated dosimetric verifications, has been described previously (18). For irradiations, mice were anesthetized using an intraperitoneal injection of ketamine (50-150 mg/kg, 100 mg/mL, Henry Schein) and xylazine (10 mg/kg, 20 mg/mL, Anased, Lloyd Laboratories).

### 2.4 Assessments of radiation-induced mucositis, body weight, and radiation-associated pain

Daily for the first 21 days after irradiation, body weights were recorded, and radiotherapy-induced oral mucositis (RIM) severity was assessed on a 5-point scale (18). Nesting and grooming activities were characterized at the time of maximal RIM severity (day 11 post-IR), and after RIM resolution (day 19 post-IR), as indicators of RAP severity (see **Supplemental Materials** for information related to assay rationale and design). These assessments were made by a single, unblinded observer (CSM).

### 2.5 Tumor cell inoculation

Tumor cells were injected into a lateral tail vein of manually restrained unanesthetized mice on day 11 post-IR (i.e., at the time of maximal glossitis severity). A total of 2×10^5^ 4T1-Luc2 cells suspended in 0.1 mL sterile PBS were injected into BALB/c mice; and 2×10^5^ B16F10-Luc2 cells in 0.1 mL sterile PBS or 1×10^6^ MOC2-Luc2 cells in 0.2 mL sterile PBS were injected into C57BL/6 mice).

### 2.6 Tumor burden and growth

Lung tumor burden was assessed *in vivo* using bioluminescence imaging (BLI) – hereafter referred to as “transthoracic BLI” (IVIS Lumina II *in vivo* imaging system; PerkinElmer Ltd.). Dorsal thoracic fur was removed with depilatory cream and mice then underwent intraperitoneal injection of D-luciferin (150 mg/kg in 0.1 mL sterile PBS; Xenogen Corp.); 10-15 minutes later, mice were isoflurane-anesthetized and imaged in ventral recumbency (imaging parameters: 745 nm excitation and >800 nm emission filters, auto exposure time, and a binning factor of 1). For analysis, a 1.5 x 1.5 cm region of interest (ROI) was placed over the thorax. Bioluminescence was expressed as the total radiant efficiency (total flux [p/s]). Lung tumor burden was measured *ex vivo* at the end of every experiment. Mice were euthanized and the entire thoracic pluck (including tongue, trachea, esophagus, thyroid glands, thymus, heart, and lungs) was removed. To facilitate visualization of nodules in 4T1-Luc2 and MOC2-Luc2 bearing mice, 3 mL of 15% India ink (diluted with PBS, pH 7.4) was instilled into the lungs *via* the trachea. Lungs were immediately rinsed with unfiltered non-sterile distilled water, and then fixed overnight in 1.5 mL of Fekete’s solution (composition: 300 mL of 70% ethanol, 30 mL of 37% formaldehyde, and 5 mL of glacial acetic acid). In B16F10-Luc2 tumor bearing mice, 3 mL of Fekete’s solution was instilled into the lungs, which were then rinsed and fixed, as above. Photographs of each lung lobe’s surface were taken using a mobile phone (Galaxy S10, Samsung) and a standard lighting and background setup; tumor burden was quantified by manually counting the total number of pulmonary nodules (data expressed as number of solitary pulmonary nodules per mouse). In experiments with nodules that were too numerous to count, photographs were exported to ImageJ software (version 1.53e, National Institutes of Health), and converted to 8-bit images. The “freehand selection” tool was used to contour lungs and remove background; a ROI was drawn around each lobe and manual thresholding was used to contour tumor nodules on the images, filling the nodule shape in “red”, and clearing the background in “white” colors. The percent of the surface occupied by tumor nodules was expressed as total area fraction per mouse.

### 2.7 Modulation of ambient temperature

Standard mouse housing temperatures (22-24°C) induce environmental stress that can alter tumor growth (19, 20). To help establish the translational relevance of our work, and ensure observations were not dependent upon this form of environmental stress, we investigated the effect of standard (STDt) versus thermoneutral (TNt) housing temperature on the growth of 4T1-Luc2 tumors in female BALB/c mice, after either lingual or sham irradiation. Mice in the STDt group were housed at room temperature (21-22°C, cages placed on standard steel shelving). For the TNt group, cage warming was achieved by placing a 10” x 20.5” heat mat (iPower Seed Starter Heat Mat) underneath a standard mouse cage. Based on pilot work (see **Supplemental Materials** file), the heating pad thermostat was set to 25.6°C, which produced an average cage floor (bedding) temperature of approximately 31°C. Cage floor temperature was monitored daily using a digital thermostat controller (iPower Digital Heat Mat Controller). Mice were acclimatized to this temperature for 7 days before starting the experiment.

### 2.8 Selective chemical ablation of transient receptor potential vanilloid 1 expressing neurons

TRPV1-expressing sensory neurons are the predominant noci-receptive sensory fiber in mice, and activation of these fibers leads to pain (21, 22). In pilot work (unpublished), we found that ablation of TRPV1-expressing neurons both reduced the severity of glossitis and associated pain. Ablation was achieved using systemic administration of resiniferatoxin (RTX), a potent TRPV1 agonist (23). Here, we investigated whether RAP-enhanced tumor progression could be prevented by ablation of TRPV1-expressing sensory neurons using RTX treatment. Pharmacologic grade RTX and excipient were kindly provided by Dr. Alexis Nahama (Sorrento Therapeutics). Female BALB/c mice received either a single interscapular subcutaneous dose of RTX (300 µg/kg, 200 µg/mL) (24), or an equivalent volume of the excipient 6 days before IR or sham IR (27 *vs.* 0 Gy), and were injected (via tail vein) with 4T1-Luc2 cells as described above. Mice were randomly allocated to one of four treatment groups: 1) 0 Gy + RTX excipient (referred as “SHAM-EXCIPIENT”); 2) 27 Gy + RTX excipient (“IR- EXCIPIENT”); 3) 0 Gy + RTX (“SHAM-RTX”); or 4) 27 Gy + RTX (“IR-RTX”). To confirm successful ablation of TRPV1 neurons in the trigeminal pathway, mice were assessed weekly *via* topical administration of 0.01% capsaicin into the left eye (20 µL in approximately 26°C 0.9% sterile saline). Responses were video recorded and later reviewed by a blinded observer who counted and recorded the number of facial wipes over 1 minute immediately following topical application of capsaicin. A 50% reduction from baseline in the number of wipes per minute constituted a successful ablation.

### 2.9 Statistical analysis

Pilot studies (n=8 mice per group) were performed to calculate the expected effect and sample size (JMP 14.1; SAS Institute Inc., Cary, NC, USA), with α=0.05 and 80% power; calculated sample sizes for each experiment are listed in **Table 1**. All statistical tests were performed using commercial software (Prism version 6, GraphPad Software, Inc., La Jolla, CA). Normality was assessed using the Shapiro-Wilk normality test. An unpaired t-test (2-tailed), or 2-way repeated measures ANOVA analysis, was used for normally distributed data; p-values were adjusted for multiple comparisons using Tukey’s or Sidak *post hoc* test. The Kaplan-Meier method and the Log-rank test were used for survival analysis. P-values less than 0.05 were considered significant.

**Table 1:** Calculated sample sizes for each experiment.

List of experiment	n per experimental group	Total n/experiment	Total n used
Measurement of RAP in female BALB/c mice using a single irradiation fraction (**Supplemental Data**)	16	32	32
Measurement of RAP in female C57BL/6 mice using a single irradiation fraction (**Supplemental Data**)	20	40	39
Tumor-growth promoting effect of RAP in breast carcinoma tumor-bearing female BALB/c mice (4T1-Luc2 model)	20	40	30
Tumor-growth promoting effect of RAP in breast carcinoma tumor-bearing male BALB/c mice (4T1-Luc2 model)	12	24	22
Tumor-growth promoting effect of RAP in breast carcinoma tumor-bearing female BALB/c mice (4T1-Luc2 model); local *vs.* total body *vs.* sham irradiation	12	36	36
Tumor-growth promoting effect of RAP in breast carcinoma tumor-bearing female BALB/c mice (4T1-Luc2 model) after manipulating cage temperatures	8	32	31
Tumor-growth promoting effect of RAP in melanoma tumor-bearing female C57BL/6 mice (B16F10-Luc2 model)	20	40	39
Tumor-growth promoting effect of RAP in oral carcinoma tumor-bearing female C57BL/6 mice (MOC2-Luc2 model)	16	32	30
Tumor-growth promoting effect of RAP in oral carcinoma tumor-bearing female C57BL/6 mice (MOC2-Luc2 model); survival assessment	16	32	29
Effects of systemic ablation of TRPV1-expressing neurons on tumor growth in breast carcinoma tumor-bearing female BALB/c mice (4T1-Luc2 model)	20	80	73

## 3 Results

### 3.1 Single fraction lingual irradiation is associated with mucositis and pain

Single-fraction 27 Gy irradiation of the rostral tongue of BALB/c mice causes mild (grade 1) glossitis 8 to 9 days post-IR, with moderate-to-severe (grade >2) glossitis at days 10 to 12 post-IR (**Supplementary Figure 1C**). As compared with SHAM controls, severe glossitis was associated with >15% reductions in body weight (**Supplementary Figure 1D**) and with measures of severe acute RAP; IR mice demonstrated decreased ability to shred nest material, and decreased ability to adequately build nests (**Supplementary Figure 1E**); they also had diminished grooming (**Supplementary Figure 1F**). All behaviors normalized by day 19 post-IR. Identical changes were seen in female C57BL/6 mice with RIM >2 (**Supplementary Figures 1G–J**).

### 3.2 Lung tumor burden in BALB/c mice is significantly increased when 4T1-Luc2 cells are intravenously injected at the time of severe mucositis and pain

**Figure 1:**
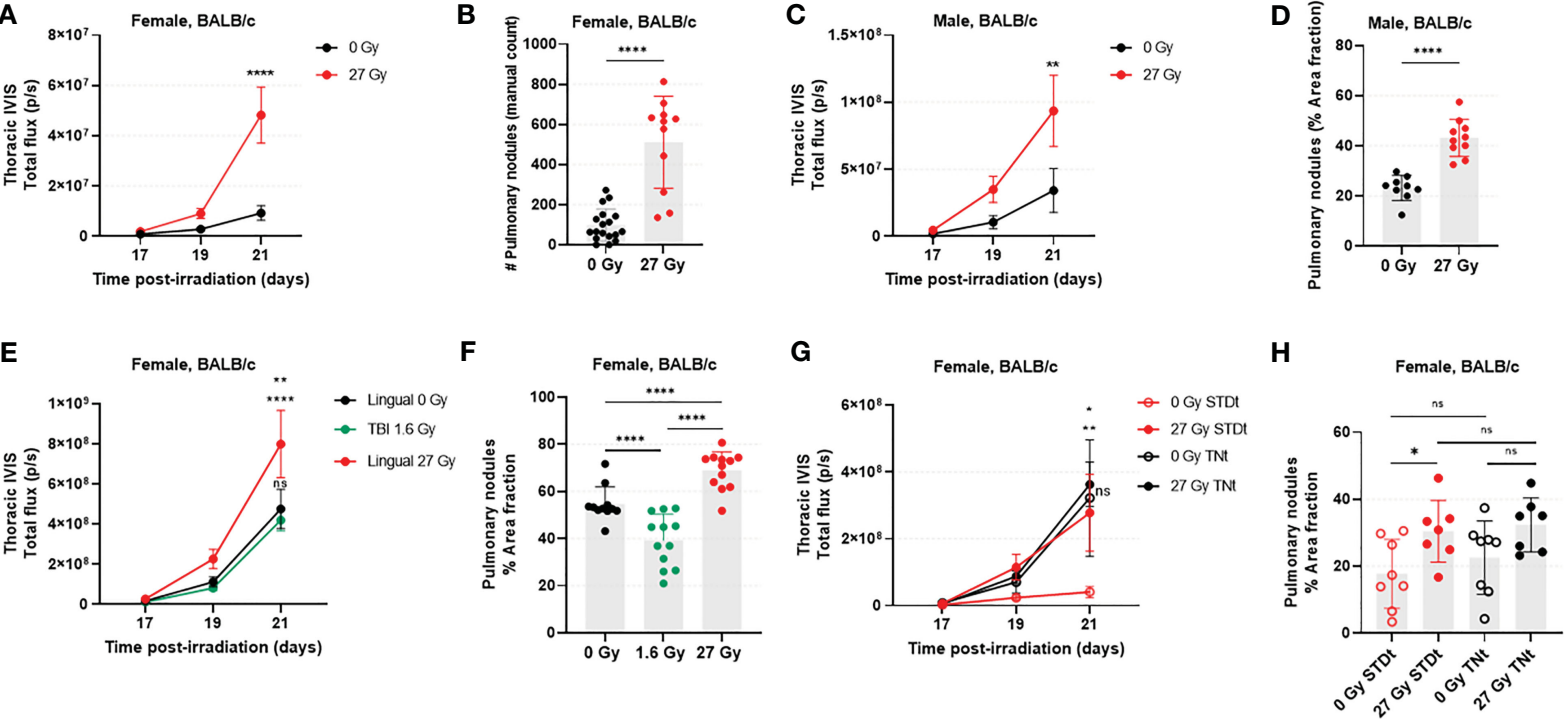
RAP is associated with rapid growth of 4T1-Luc2 cells following tail vein injection in the syngeneic host. In female mice (n=12-18/group), transthoracic BLI **(A)** and *ex vivo* manual count **(B)** were significantly higher in the irradiated mice. These findings were recapitulated in male mice (n=11/group), where transthoracic BLI **(C)** and number of surface pulmonary nodules **(D)** were significantly higher in tongue-irradiated mice. The experiment was then repeated in female mice, with addition of a second control group in which mice underwent total body irradiation (TBI) to assess the effects of off-target radiation to non-lingual tissues. In this study (n=12 mice/group) we found a significant increase in transthoracic BLI for irradiated versus control mice; there was no detectable statistical difference between the two control groups **(E)**. Likewise, IR mice had more lung tumors as compared to SHAM and TBI mice. Tumor burden was markedly decreased in the TBI versus SHAM group **(F)**. We also evaluated the effects of cage temperature (n=7-8/group). Results **(G, H)** were as expected for standard (STDt) housing conditions, recapitulating what was already described in A through **(F)** However, when housed in warm cages (TNt), there was no detectable difference in lung tumor burden for IR and SHAM mice, and the tumor burden was higher for both groups than it was for the STDt-SHAM mice. Data for BLI imaging are presented as mean ± standard error of mean (SEM), and data from post-mortem evaluations are presented as mean ± standard deviation (SD). *p<0.05, **p<0.01; ****p<0.0001; ns., not significant.

Pulmonary luciferase activity was always measurable by day 17 post-IR. Signal intensity increased until day 21, when tumor burden was significantly greater in female IR mice versus SHAM (5.2-fold increase, p<0.0001; **Figure 1A**). Similar results were observed in males; by day 21, irradiated males had a 2.7-fold increase in transthoracic BLI compared with controls (IR *vs.* SHAM, p=0.0079; **Figure 1C**). Imaging results (tumor burden) were substantiated *via* post-mortem (*ex vivo*) evaluation (IR *vs.* SHAM, female p<0.0001, male p<0.0001; **Figures 1B, D**).

### 3.3 Off-target radiation does not increase the burden of 4T1 lung tumors

Transthoracic BLI results revealed that tumor burden was statistically significantly greater in IR mice than in SHAM (1.8-fold increase, p=0.0039) and TBI (1.9-fold increase, p=0.0008) mice at day 21 post-IR (**Figure 1E**). Furthermore, tumor burden was no different between SHAM and TBI groups at any timepoint (p>0.05; **Figure 1E**). We confirmed these results post-mortem by calculating and comparing the percent area of lung covered by visible nodules (**Figure 1F**). IR mice had significantly more 4T1-Luc2 tumor nodules as compared to SHAM mice (IR *vs.* SHAM, p=0.0002; IR *vs.* TBI, p<0.0001). Interestingly, in this analysis, there was an apparent reduction in lung tumor burden in the mice having undergone TBI as opposed to sham irradiation (p=0.0009).

### 3.4 Standard (subthermoneutral) housing conditions do not appear to enhance 4T1-Luc2 lung tumor burden

This experiment was designed to assess whether thermoneutral environmental temperatures would eliminate the apparent tumor promoting effects of high-dose tongue irradiation (e.g., by reducing environmental stress and thus potentially restoring an effective host response against tumor growth). Here, we were able to replicate our prior experimental finding that with standard mouse housing conditions, tongue irradiation is associated with increased tumor burden in the 4T1-Luc2 model (6.7-fold increase; IR- STDt *vs.* SHAM-STDt, p=0.0028; **Figures 1G, H**). Warming the cages enhanced tumor burden in SHAM mice (7.8-fold increase; SHAM-STDt *vs.* SHAM-TNt, p=0.0271), but contrary to our expectation, tongue irradiation had no impact on the burden of 4T1-Luc2 tumors when mice were housed in thermoneutral temperature conditions (1.1-fold increase: IR-TNt *vs.* SHAM-TNt, p=0.9800; and 1.3-fold increase: IR-STDt *vs.* IR-TNt, p=0.6599). Tumor burden rapidly increased in all IR and SHAM mice housed under thermoneutral housing conditions, and their tumor progression was more similar to the one observed in IR-STDt mice. We confirmed these results by calculating the percent area of lung covered by visible nodules (*ex vivo*), however, here we did not observe a statistically significant difference between SHAM groups (STDt *vs.* TNt, p=0.3841).

### 3.5 Tongue irradiation does not increase the burden of B16F10-Luc2 lung tumors, but was associated with early death in MOC2-Luc2 bearing mice

**Figure 2:**
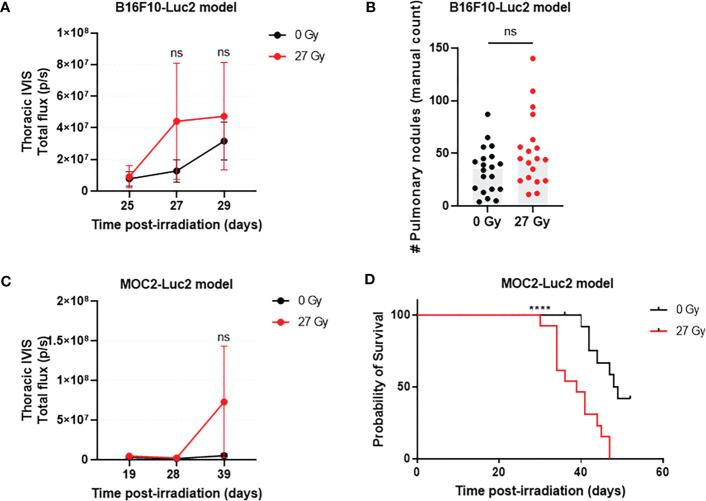
The effect that acute RAP has on distant tumor growth varies with tumor model. In the B16F10-Luc2model (n=19-20/group) there were no significant differences in lung tumor burden (transthoracic BLI) between IR and SHAM C57BL/6 mice at days 25, 27, and 29 post-injection **(A)** or by post-mortem counting **(B)**. Similarly, there was no detectable difference in transthoracic BLI strength in C57BL/6 mice with MOC2-Luc2 tumors (n=13-16/group) **(C)**. However, pulmonary metastasis-attributable deaths occurred earlier for IR mice versus SHAM **(D)**. Data are presented as mean ± standard deviation (SD). ****p<0.0001; ns., not significant.

In the B16F10-Luc2 model, transthoracic BLI signal appeared to increase at 27 and 29 days post-IR, but there was no difference in tumor burden between IR and SHAM mice at any timepoint (**Figure 2A**). Based upon postmortem evaluation, tongue irradiation caused a non-significant 1.5-fold increase in lung tumor burden versus sham irradiation (**Figure 2B**). In the MOC2-Luc2 model, transthoracic BLI signals were first detected at day 19 post-IR in all mice, but there was no further increase in signal intensity on days 28 and 38 post-IR (**Figure 2C**). We did however observe increased respiratory effort and early death in some of the IR mice; we therefore repeated this experiment using a new cohort and monitored respiratory status and survival. IR mice started to display progressive respiratory effort 30 days post-IR, leading to death in all cases. By contrast, only a few SHAM mice developed mild respiratory effort at this point, and almost all survived to day 52 (end of study) when all mice were euthanized. Pulmonary nodules (determined by visual inspection of lungs) were found in all mice at the time of death. The median overall survival time of IR mice was 39 days (95% CI: 30 to 47 days), and 48.5 days (95% CI: 36 to 52 days) for SHAM mice (p=0.0002; **Figure 2D**).

### 3.6 The growth-promoting effects that tongue irradiation had on 4T1 lung tumors is mitigated by systemic ablation of TRPV1 sensory nerves

**Figure 3:**
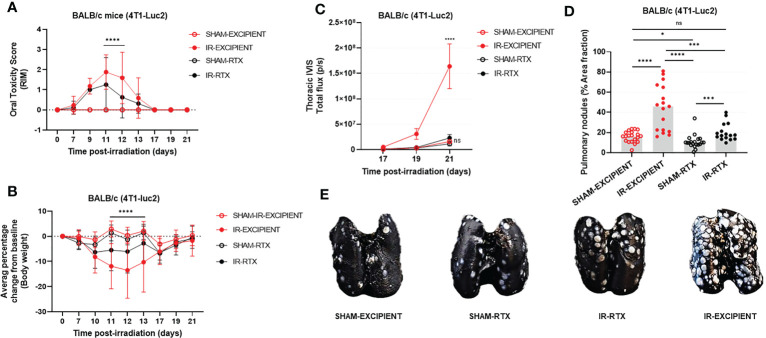
Systemic ablation of TRPV1-expressing neurons reduces the severity of RIM, and mitigates the tumor-promoting effects of RAP. In all mice, tongue irradiation caused glossitis **(A)** that was accompanied by weight loss **(B)**. The severity of these changes was reduced with pre-irradiation resiniferatoxin treatment. Resiniferatoxin pre-treatment also reduced the tumor-promoting effect of high-dose tongue irradiation as measured *in vivo* using transthoracic BLI **(C)** and postmortem enumeration of nodules **(D)**. Representative images of the India-ink treated lungs used for manual nodule enumeration are depicted in **(E)**. Data are presented as mean ± standard deviation (SD); *p<0.05; ***p<0.001; ****p<0.0001; ns., not significant.

Neither RTX nor excipient caused glossitis or weight loss in SHAM animals (**Figures 3A, B**). By days 10 to 13 post-IR, the vast majority of IR-EXCIPIENT mice had developed RIM scores >2 and profound weight loss (>15%). By contrast, varying degrees of RIM were found in IR-RTX mice (RIM 0: ~44%, RIM 1: ~19%, and RIM >2: ~38%; mean of 1.25, standard deviation of 1.3; data from day 11 post-IR data). In these animals, weight loss was minimal (<5%).

Overall, RTX treatment reduced tumor burden in IR mice to levels similar to what was observed in the SHAM groups. As expected, and based upon aforementioned experimental results, IR-EXCIPIENT mice had a 10-fold higher mean transthoracic BLI signal as compared with SHAM-EXCIPIENT at day 21 post-IR (p<0.0001; **Figure 3C**). There was minimal increase in transthoracic BLI signals in IR-RTX mice compared to SHAM-RTX animals (2-fold increase, p=0.9155; **Figure 3C**), and furthermore, transthoracic BLI signal revealed that RTX markedly reduced tumor burden in irradiated mice (IR-RTX *vs.* IR-EXCIPIENT, 7.5-fold decrease, p<0.0001; **Figure 3C**). Importantly, RTX treatment itself had no measurable impact on tumor burden as measured by transthoracic BLI in sham-irradiated mice (SHAM-RTX *vs.* SHAM-EXCIPIENT, p=0.9933; **Figure 3C**).

We confirmed these results in post-mortem evaluations (**Figures 3D, E**). A statistically significant difference was observed between IR and SHAM excipient-treated mice (p<0.0001), and between excipient and RTX-treated IR mice, with smaller tumor burden detected in the RTX group (p=0.0004). There was a slight yet significant reduction in tumor burden in SHAM-RTX mice compared to SHAM-EXCIPIENT mice (p=0.0137), and a significant increase in tumor burden in IR-RTX mice as compared with SHAM-RTX mice (p=0.0008).

## 4 Discussion

In humans, pain from cancer (or cancer treatment) can be a clinical bellwether for cancer prognosis. Severe pain is strongly associated with poor survival in pancreatic adenocarcinoma, and pain predicts disease progression in castration-resistant prostate cancer (25, 26). Experimental work has shown that pain at one site can enhance tumor progression at distant sites (14). For many cancers, tumor behavior is influenced by the nervous system, and direct communication between nerves and cancer cells can promote tumor growth and metastasis (27, 28). Increasingly, it is recognized that molecules involved in pain signaling are also involved in cancer progression, and beyond interactions in the local tumor microenvironment, activation of certain neuronal signaling pathways by the same molecules can also facilitate metastatic cancer progression (29, 30). Pain is a common complication of definitive radiotherapy, especially at certain anatomic sites, such as breast, head, and neck. Most patients undergoing definitive head and neck irradiation will struggle with treatment-associated pain (9, 10) that is poorly responsive to available analgesics (31); unfortunately, 30-40% of treated patients will eventually fail locally or develop metastasis, but it’s unknown whether RAP might contribute to those oncologic failures (32). Here, we demonstrate that in an experimental animal model of lung metastasis, severe acute orofacial RAP is associated with rapid tumor progression, and that effect can be mitigated *via* chemical ablation of sensory neurons which transmit noxious signals.

Our experimentation began with the 4T1-Luc2 model because it was accessible and tumor growth is rapid; strong and stable transfection of the luciferase reporter, paired with a syngeneic host that has unpigmented skin, meant that lung tumors could be reliably visualized *in vivo* using BLI. Prior tongue irradiation strongly promoted lung tumor growth after tail vein injection, especially in female mice, and we thus utilized this model system for most subsequent experiments. Tongue irradiation and pain did not affect growth of B16F10-Luc2 cells in C57BL/6 mice, suggesting that the phenomenon may be tumor type dependent. However, the apparent tumor-promoting effects of tongue irradiation (and pain) were recapitulated in the MOC2-Luc2 model, which is important because it helps establish the translational relevance by linking a complication of oral irradiation with altered growth of an oral cancer cell line (33). Our experiments did not assess animals with spontaneously occurring tumors, and our system for studying the effects of local tissue irradiation on distant tumor growth is limited by failure of the tail vein injection model to recapitulate all steps of the metastatic cascade (34).

Kaplan and Murphy (1948) described an increase in risk of early metastasis amongst patients undergoing radiotherapy for epidermoid carcinomas (35), and Strong et al. (1978), reported a correlation between primary tumor irradiation and higher incidence of metastasis in head and neck cancer patients (36). Similar reports exist for other cancers, and despite these reports, and relevant experimentation, the science remains unclear. If radiation does promote metastasis, this seems most likely with subtherapeutic radiation dosing. However, firm conclusions cannot be made based upon the existing evidence. Our experiments were designed to address two methodologic concerns of prior research. First, Repasky et al. (20) have shown that standard mouse housing temperatures are about 10°C lower than the temperature at which mice expend no excess energy keeping themselves warm or cool. Housing in subthermoneutral conditions produces metabolic stress that limits the translational value of standard mouse tumor experiments (19, 20). The current thinking is that this stress, mediated by beta adrenergic signaling, allows tumor escape from immune surveillance, and comes with risk of exaggerating tumor growth and treatment effects. Thus, we expected that performing our experiments under thermoneutral conditions (~30°C) would ameliorate weight loss that mice otherwise display when experiencing severe glossitis, and that we would also see slower tumor growth kinetics. By contrast, we observed more severe weight loss and behavioral impairments indicative of pain (data not shown), and tumor growth was actually accelerated. Our work is limited by the fact that core body temperature was not monitored. Nonetheless, these results support the idea that our experimental results were not a “false positive” signal as a result of thermal stress caused by standard housing conditions. Second, with our single fraction 27 Gy tongue irradiation protocol, there is ~1.6 Gy absorbed by the body; this total body exposure is insufficient to induce oral mucositis, pain behaviors, or significant changes in expression of the ion channels that regulate RAP (18). However, that amount of radiation dose may be sufficient to alter immune function. Our data demonstrate that a 1.6 Gy total body exposure does not enhance lung tumor growth. Interestingly, our data actually show the opposite: mice having undergone TBI actually had reduced lung tumor burdens (*vs.* sham-irradiated mice). It’s possible that the prior exposure of the lungs to low dose radiation may have had anti-inflammatory effects that were protective against tumor growth (37).

To establish direct evidence that accelerated lung tumor growth was attributable to pain, we sought to demonstrate that this observed effect was reduced when pain was reduced. However, as in humans, murine orofacial RAP is difficult to treat; our unpublished data indicate minimal to no benefit from non-steroidal anti-inflammatory drugs, opioids, or a combination thereof. As an alternative, we used RTX, which is a potent analgesic by virtue of its ability to cause rapid and widespread ablation of TRPV1-expressing noci-receptive sensory neurons (38). Additionally, it’s been estimated that a reasonable proportion (about 30%) of sensory neurons innervating the tongue express TRPV1 (22, 25, 39, 40), and TRPV1 appears to be an important regulator of orofacial RAP (18). Here, we found that a single high-dose of RTX led to substantial and lasting desensitization to a TRPV1 agonist (capsaicin), and when given before radiation, RTX led to reductions in both glossitis severity and RAP behaviors (grooming and nesting). The provision of RTX also abrogated the tumor-promoting effects of tongue irradiation that we’d previously observed in the 4T1-Luc2 model. Whilst these results support our hypothesis, it is worth considering that RTX was administered systemically. TRPV1 receptors are widely expressed in afferents innervating airways and lungs (41), which points to the possibility that non-analgesic mechanisms may explain some or all the effect we measured. While RTX has previously been shown to reduce tumor growth in certain non-painful models of cancer (42), in the syngeneic 4T1-Luc2 model, it appears that RTX accelerates early tumor growth *via* increased vascular leakage (43). Despite these concerns, here we robustly showed reduced TRPV1 neuron activity decelerates the growth of tumors at sites distant from where RAP is generated.

Several limitations should be considered. First, the work presented herein relied upon RAP emanating from the oral cavity. The biology underlying RAP which develops after both oral cavity and hindquarter irradiation is similar in that TRP channels seem to play an important role at both anatomic sites (18, 44); nonetheless, it will be important in future work to confirm whether or not RAP from extra-oral sites and/or RAP of lesser severity has similar tumor-promoting effects. Second, our approach of using large single fractions (27 Gy) differs substantially from clinically utilized protocols which most often deliver much higher total doses (e.g., 50 Gy or more) in much smaller fractional doses (approximately 2 Gy per fraction). While our experimental irradiation caused mucositis, weight loss and pain that is similar to the morbidity experienced by human patients undergoing head and neck cancer irradiation, it remains uncertain how the neurobiological impacts of our experimental irradiation protocol might compare with a more clinically relevant protocol. For that reason, future work should seek to understand whether these effects remain significant with fractionated radiation, and in the setting of combinatorial therapies (e.g., chemoradiotherapy). It will also be important to understand whether there are radiation dose-modifying effects of RAP on primary tumor control.

## 5 Conclusions

This body of research provides experimental evidence suggesting that acute RAP may contribute to early death by hastening lung tumor growth in a mouse model of tail vein induced pulmonary metastasis. We demonstrated that both male and female mice develop more lung tumors when 4T1-Luc2 murine breast carcinoma cells are intravenously injected into their syngeneic host at the time of maximally severe tongue RAP. Similarly, MOC2-Luc2 lung tumor-attributable death was hastened when tumor cells were injected at the time of maximally severe RAP. Our experimental data clearly indicate that this accelerated tumor growth was not a result of off-target radiation, nor was it an artefact of the environmental stress caused by standard animal housing temperatures. This points towards tongue tissue irradiation as the cause for accelerated growth of tumors in the lungs, and because lung tumor growth rates are normalized when RAP was reduced *via* RTX treatment, we conclude that the effect is related to radiation-induced neuronal activation and/or signaling.

## Data availability statement

The raw data supporting the conclusions of this article will be made available by the authors, without undue reservation.

## Ethics statement

The animal study was reviewed and approved by Institutional Animal Care and Use Committee at North Carolina State University (protocol #’s: 18-061-B and 19-810-B).

## Author contributions

Study design: MN, BL, and SM. Data collection: CM, EG, KM, YG, and YL. Data analysis and interpretation: EG, CM, MN, and BL. Writing of the manuscript: CM, MN, and BL. Revision of the manuscript: All authors. Statistical analysis: CM. All authors contributed to the article and approved the submitted version.
