# Supplementary material for: Acute radiotherapy-associated oral pain may promote tumor growth at distant sites
Source: Front Oncol. 2023 May 19;13:1029108. doi: 10.3389/fonc.2023.1029108 (PMC10235601; doi:10.3389/fonc.2023.1029108)
Supplement: Supplementary file 1 [file DataSheet_1.docx]

Supplementary Material

**Supplemental data**

1. Assessment of radiation associated mucositis and pain

Observations in previous experiments (unpublished) indicated that normal laboratory rodent behaviors, such as nesting and grooming, were perturbated in mice that had been irradiated and developed RIM/RAP. Both activities are highly dependent on mouth and tongue function (i.e., to shred the material or licking the forepaws, respectively), therefore, we decided to use assessments of how well these activities were performed as markers of pain. To our knowledge, no prior work has evaluated nesting and grooming behaviors in the context of oral pain in mice, therefore, we adapted published protocols (1, 2). Briefly, mice were transferred to individual testing cages containing one commercially available cotton fiber nestlet (5 cm x 5 cm, 5 mm thick, approximately 2.5 g each), food and water. After 12 hours (dark cycle), nesting (i.e., building activity) and grooming (i.e., physical appearance evaluation) activities were evaluated by a trained, unblinded evaluators using a predefined scoring system. Briefly, nesting activity was measured using a 3-point scale (See illustrations in Supplemental Figure 1A): 0 (zero) – Most of the nestlet has been completely torn into small pieces; pieces arranged together into one area of the cage; 1 – Incomplete shredding of the nestlet; large pieces may remain and may remain scattered around the cage (not all arranged together); and 2 – nestlet was not noticeably touched. Self-grooming behavior was categorized using a 4-point scale (See illustrations in Supplemental Figure 1B): 0 (zero) – Clean, smooth haircoat; 1 – Fur on body begins to look messy, but face remains normal; 2 – Face and body look messy, with hair “standing on end”; and 3 – Disheveled; wet/greasy appearance, possible discoloration of coat, erythema (reddened skin), and areas of hair loss (alopecia).

1. Cell lines preparation and protocol optimization

In previous pilot studies, we established three murine lung tumor models by intravenously injecting 4T1-Luc2, B16F10-Luc2 or MOC2-Luc2 cell lines into either BALB/c or C57BL/6 mice, monitoring cancer progression *in* *vivo* and *ex* *vivo*. Mice either received a single high-dose of lingual irradiation (27 Gy) or sham irradiation (0 Gy). All tumor cell lines were inoculated 11 days after irradiation (peak of glossitis severity). Two pilot studies of 8 mice each determined optimal dosage of 4T1-Luc2 cells, as well as injection route, *in* *vivo* imaging protocol, *ex* *vivo* lung staining technique and quantification of lung’s tumor burden. Dose was defined based on published information for that tumor model (3): 2×10^5^ 4T1-Luc2 cells/mouse suspended in 0.1 mL of sterile PBS were injected intravenously (sterile 26-guage needle) into female BALB/c mice. Tumor growth was monitored *in* *vivo* using bioluminescence imaging (BLI) by taking images at days 7, 14, 17, 19 and 21 post-injection. The first pilot evaluated a retro-orbital injection approach, showing a progressive growth of pulmonary tumors (expressed as transthoracic BLI total/flux signal) within the first week after injections, starting at day 17 post-IR. A comparable tumor-burden difference was found between both groups at day 21 post-IR. At this timepoint, mice started to develop signs of body weight loss and respiratory disease (pulmonary assessment of advanced metastasis (PAAM) technique) (4). Mice were euthanized on day 21 post-IR, and lungs were removed. An *ex* *vivo* tumor burden evaluation was performed by counting the number of nodules that were visible on the lung’s surface. Here we established that 3 mL of 15% ink solution (diluted with PBS) and later fixation of lungs using a formalin-based solution (Fekete’s solution) was sufficient to highlight these growths within all lung’s lobes (white nodules on a black background). Exophthalmia, as a result of local tumor growth after injections, was a common challenge in this pilot. A second pilot assessed the tail vein injection (TVI) approach. Similarly, lung tumor growth initiated in all mice at day 17 post-IR (6 days post-injection), and differences between groups were significant at day 21 post-IR (*in* *vivo* BLI analysis). There were no major complications, and we therefore decided to select the TVI approach for subsequent experiments. For the B16F10-Luc2 and MOC2-Luc2 models, two consecutive pilots (n=8/each) for each model were performed to determine optimal dosage, injection route, *in* *vivo* imaging protocol, *ex* *vivo* lung staining technique and quantification of lung tumor burden. Briefly, mice were inoculated 11 days after irradiation via tail vein injections with either B16F10-Luc2 (2×10^5^ cells/mouse suspended in 0.1 mL of sterile PBS) or MOC2-Luc2 cells (1×10^6^ cells/mouse suspended in 0.2 mL of sterile PBS). Inoculation doses were established from previous work (5, 6). We first observed lung bioluminescence in the B16F10-Luc2 and MOC2-Luc2 injected mice at day 25 and 19 post-IR, respectively. At euthanasia, visible tumor nodules were found in the lungs of most mice. Only MOC2-Luc2 cells were stained with India ink and de-stained with Fekete’s solution. B16F10-Luc2 cells did not require staining because their melanin pigmentation allowed easy visualization on unstained lungs; therefore, lungs were inflated using Fekete’s solution, and later stored in the same solution.

1. Pilot study to establish methods for modulation of cage temperature

A series of pilot experiments were performed to determine how to modify housing temperature conditions of adult mice to create cage floor temperatures of 30 to 31ºC (previously associated with thermoneutrality in mice; reference #7). First, we determined the temperatures of standard mouse cages without animals. We measured the floor temperature of 20 standard mouse cages using a 4-channel rapid read thermometer (General Tools DT4947SD thermocouple thermometer). All cages were kept inside our mouse colony at standard room temperature (21-22ºC), placed on steel shelving units, and filled with a normal amount of bedding and no mouse behavioral enrichment material. Five cage floor “zones” were assessed: the center, and each of the four corners. Temperature was recorded three times a day (8 AM, 2 PM, and 6 PM), each day for a week. In general, all zones were a similar temperature, ranging from 21.5°C to 22.7°C, with an average of 22.1°C (standard deviation of 0.27). No differences were found between the different times of the day, nor between days. Then we measured temperatures of all zones in a single cage with bedding containing adult CD1 mice (n=4/cage). After an hour with mice in the cage, temperatures ranged from 22.4°C to 24.5°C, with an average of 23.5°C (standard deviation of 0.87). In a separate experiment, we measured temperatures of 4 cages (bedding only; no animals) placed atop a 10-inch x 20.5-inch heat mat (iPower Seed Starter Heat Mat) with the thermostat set at 25.6°C. After 90 minutes, zone temperatures ranged from 29°C to 32°C, with an average of 30.5°C (standard deviation of 3.5). Ninety minutes after adding 4 CD1 adult mice to each cage, we found that temperatures ranged from 29.5°C to 32.5°C, with an average of 31°C (standard deviation of 2.3). Body temperature of the mice was not measured.

**Supplemental Figures**


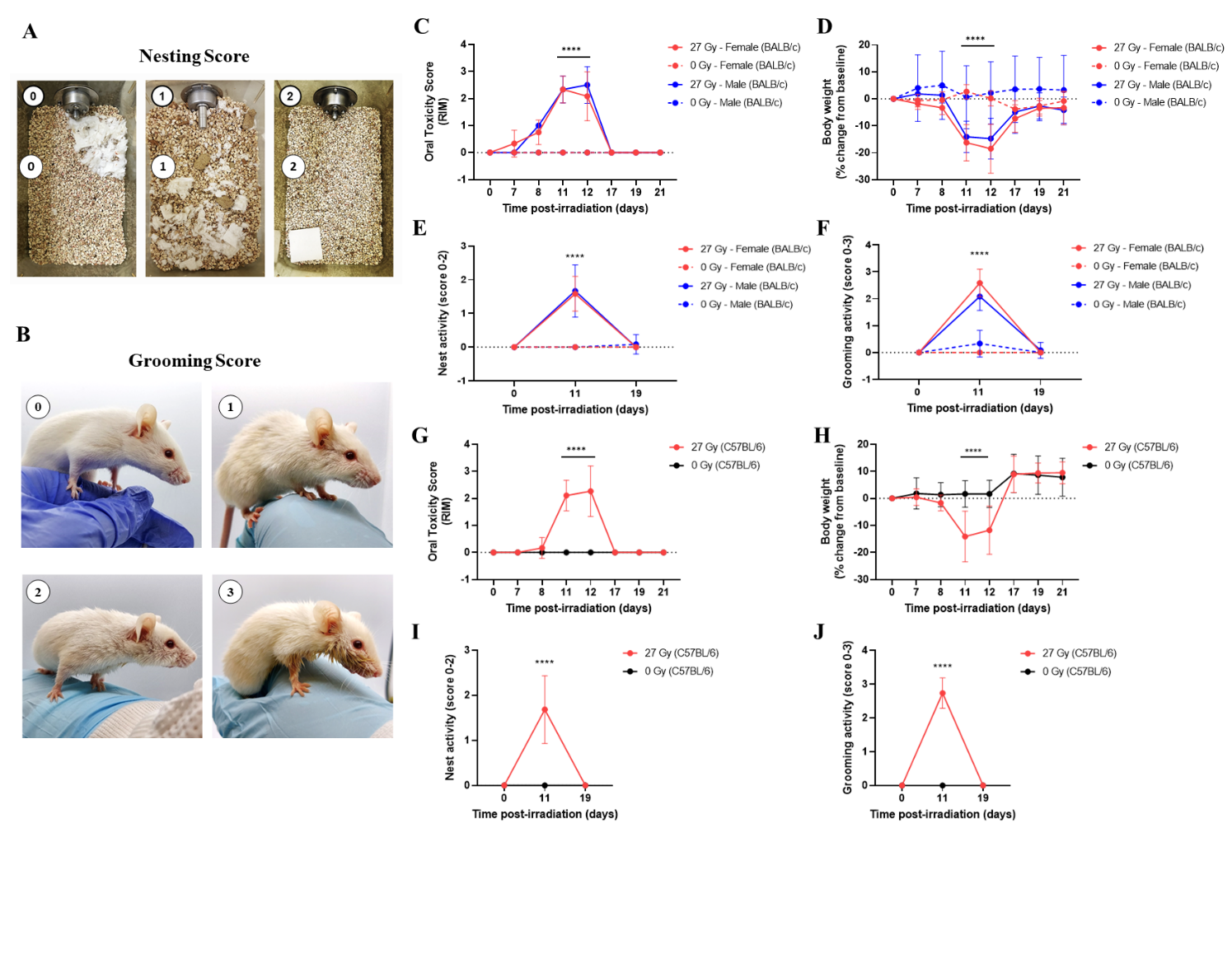


**Supplementary Figure 1: Tongue irradiation in BALB/c and C57BL/6 mice results in RAP. (A)** Nesting activity was measured using a 3-point scale: 0 (zero) – Most of the nestlet has been completely torn into small pieces; pieces arranged together into one area of the cage; 1 – Incomplete shredding of the nestlet; large pieces may remain and may remain scattered around the cage (not all arranged together); and 2 – nestlet was not noticeably touched. **(B)** Self-grooming behavior was categorized using a 4-point scale: 0 (zero) – Clean, smooth haircoat; 1 – Fur on body begins to look messy, but face remains normal; 2 – Face and body look messy, with hair “standing on end”; and 3 – Disheveled; wet/greasy appearance, possible discoloration of coat, erythema (reddened skin), and areas of hair loss (alopecia). **(C)** All tongue-irradiated BALB/c mice (female and male) experienced severe glossitis (p<0.0001), **(D)** with significant (>15%) reductions in body weight from baseline (p<0.0001), **(E)** as well as impaired nesting (p<0.0001), and **(F)** grooming (p<0.0001) activities at day 11 post-irradiation (27 Gy), as compared to sham-irradiated controls (0 Gy). **(G)** In female C57BL/6 mice, single-fraction (27 Gy) irradiation of the rostral tongue caused moderate-to-severe RIM (score >2), **(H)** severe body weight loss (>15%), and impairment of **(I)** nesting and **(J)** grooming activities (IR vs SHAM, p>0.0001 for both assays at day 11 post-IR). Data presented as mean ± standard deviation (SD); *p<0.05, **p<0.01; ***p<0.001; ****p<0.0001; ns., not significant.

|   **A** |   **B** |   **C** |
| --- | --- | --- |

**Supplementary Figure 2: Resiniferatoxin reduces RAP.** In mice having undergone subcutaneous injections of resiniferatoxin (RTX) before irradiation, reductions in corneal sensitivity to topically applied capsaicin provide evidence for effective TRPV1 neuronal ablation **(A)**. Tongue irradiation caused pain that was evidenced by elevated nesting and grooming scores at the time of maximally severe glossitis; however, nesting and grooming behaviors remained normal in the RTX-treated mice, thus indicating effective RAP reduction **(B and C)**. Data presented as mean ± standard deviation (SD); *p<0.05, **p<0.01; ***p<0.001; ****p<0.0001; ns., not significant.

**References**

1. Gaskill BN, Karas AZ, Garner JP, Pritchett-Corning KR. Nest building as an indicator of health and welfare in laboratory mice. J Vis Exp. 2013;(82):51012.
2. Burkholder T, Foltz C, Karlsson E, Linton CG, Smith JM. Health evaluation of experimental laboratory mice. Curr Protoc Mouse Biol. 2012; 2:145–65.
3. Lee HS, Ha AW, Kim WK. Effect of resveratrol on the metastasis of 4T1 mouse breast cancer cells in vitro and in vivo. Nutr Res Pract. 2012;6(4):294–300.
4. Mendoza A, Gharpure R, Dennis J, Webster JD, Smedley J, Khanna C. A novel noninvasive method for evaluating experimental lung metastasis in mice. J Am Assoc Lab Anim Sci. 2013;52(5):584–9.
5. Nagaya T, Nakamura Y, Okuyama S, Ogata F, Maruoka Y, Choyke PL, et al. Syngeneic mouse models of oral cancer are effectively targeted by anti–CD44-based NIR-PIT. Mol Cancer Res. 2017;15(12):1667–77.
6. Nobumoto A, Nagahara K, Oomizu S, Katoh S, Nishi N, Takeshita K, et al. Galectin-9 suppresses tumor metastasis by blocking adhesion to endothelium and extracellular matrices. Glycobiology. 2008;18(9):735–44.
7. Repasky EA, Evans SS, Dewhirst MW. Temperature matters! And why it should matter to tumor immunologists. Cancer Immunol Res. 2013;1(4):210–6.
